# Adipose Tissue Serves as a Reservoir for Recrudescent *Rickettsia prowazekii* Infection in a Mouse Model

**DOI:** 10.1371/journal.pone.0008547

**Published:** 2010-01-01

**Authors:** Yassina Bechah, Christopher D. Paddock, Christian Capo, Jean-Louis Mege, Didier Raoult

**Affiliations:** 1 Unit for Research on Emergent and Tropical Infectious Diseases (URMITE), CNRS-IRD UMR 6236, Faculty of Medicine, University of the Mediterranean, Marseille, France; 2 Infectious Diseases Pathology Branch, Division of Viral and Rickettsial Diseases, Centers for Disease Control and Prevention, Atlanta, Georgia, United States of America; Institut de Pharmacologie et de Biologie Structurale, France

## Abstract

Brill-Zinsser disease, the relapsing form of epidemic typhus, typically occurs in a susceptible host years or decades after the primary infection; however, the mechanisms of reactivation and the cellular reservoir during latency are poorly understood. Herein we describe a murine model for Brill-Zinsser disease, and use PCR and cell culture to show transient rickettsemia in mice treated with dexamethasone >3 months after clinical recovery from the primary infection. Treatment of similarly infected mice with cyclosporine failed to produce recrudescent bacteremia. Therapy with doxycycline for the primary infection prevented recrudescent bacteremia in most of these mice following treatment with dexamethasone. *Rickettsia prowazekii* (the etiologic agent of epidemic typhus) was detected by PCR, cell culture, and immunostaining methods in murine adipose tissue, but not in liver, spleen, lung, or central nervous system tissues of mice 4 months after recovery from the primary infection. The lungs of dexamethasone-treated mice showed impaired expression of β-defensin transcripts that may be involved in the pathogenesis of pulmonary lesions. In vitro, *R. prowazekii* rickettsiae infected and replicated in the murine adipocyte cell line 3T3-L1. Collectively these data suggest a role for adipose tissue as a potential reservoir for dormant infections with *R. prowazekii*.

## Introduction

Epidemic, or louse-borne typhus, caused by *Rickettsia prowazekii*, is a potentially fatal disease that typically occurs as large, explosive outbreaks associated with war, famine, and overcrowding during periods of civil turmoil [1]–[3]. Until recently; epidemic typhus was considered a disease of the past; however it re-emerged dramatically in 1997 in Burundi where it affected an estimated 100,000 refugees of the Rwanda civil war [4]–[6]. The disease also has occurred recently as smaller outbreaks, in Russia in 1997 [7] Peru in 1998 [8], and as isolated sporadic cases in Algeria [9], [10] the United States [11] and Europe [12].

*R. prowazekii* rickettsiae can remain dormant for years or even decades in patients who recover from the primary infection [13]. In certain individuals, stress or waning immunity are likely to reactivate this persistent infection, and cause a recrudescent form of typhus known as Brill-Zinsser disease [14], [15]. Cases of Brill-Zinsser disease have been reported in the United States [16]–[18], Canada [19] and Europe [20]. Brill noted that, “the causative agent of typhus, once acquired, remains latent for many years in an indeterminable number of individuals and may become active in a fraction of these under circumstances of fading immunity” [21]; however, the precise pathophysiologic cues that reactivate infection remain unknown. Brill-Zinsser disease is considered a milder illness than classical epidemic typhus, and the case-fatality rate of non-treated recrudescent disease is approximately 1.2%–2.5% [22], [23]; nonetheless, many patients with Brill Zinsser disease are moderately to severely ill [14], [22], [23].

The anatomic and cellular locations of dormant *R. prowazekii* rickettsiae have remained a mystery since the first descriptions of recrudescent typhus by Brill nearly 100 years ago [14], [22]. By using a recently developed murine model of epidemic typhus [24], we describe an experimental model of recrudescent typhus, and identify adipose tissue as a site from which dormant *R. prowazekii* rickettsiae can be reactivated.

## Results

### Effect of Dexamethasone and Cyclosporine on R. prowazekii Reactivation in Mice

Rickettsemias of 28 BALB/c mice infected with *R. prowazekii* were determined by quantative real-time PCR (qPCR). The highest levels were observed 3 to 6 days post-infection (pi), decreased by day 9, and were non-detectable at day 30 pi. One month after no rickettsial DNA could be detected in the blood of previously infected mice (60 days post-inoculation), groups of mice were given either dexamethasone for 30 days, cyclosporine for 20 days, or PBS for 20 days. The immunosuppressing effects of these regimens were assessed by measuring IFN-γ release by mouse splenocytes harvested at the end of the immunosuppressive treatment and stimulated in vitro with heat-inactivated *R. prowazekii*. Levels of IFN-γ released from splenocytes of infected mice treated with dexamethasone or cyclosporine were significantly decreased compared to the levels in infected mice that received sham immunosuppression (table 1).

**Table 1:** Levels of interferon (IFN)-γ secreted by murine splenocytes following treatments with dexamethasone or cyclosporine.

	IFN-γ content (pg/ml)	
Treatment	Stimulated	Not stimulated
PBS control	1146±64	355±34
Cyclosporine	117±31	81±18
Dexamethasone	72±12	20±5

A transient bacteremia, (mean = 73 DNA copies/µL blood, range = 12 to 118 copies/µL blood) was detected by using qPCR in 7 (88%) of the 8 dexamethasone-treated mice 3 days after the completion of treatment, and disappeared by 8 days (table 2). The amount of rickettsial DNA varied by tissue type, and ranged from 14 to 147 copies/mg tissue in livers, 234 to 317 copies/mg tissue in lungs, 420 to 705 copies/mg tissue in brains, and from (24 to 1750 copies/mg tissue in spleens) (table 2). DNA of *R. prowazekii* remained detectable in several of these tissues 8 days following the completion of dexamethasone treatment (1/3 mice in liver and spleen and 3/3 mice in lung and brain). DNA of *R. prowazekii* was not detected by qPCR in the blood of any cyclosporine-treated or sham-immunosuppressed mouse.

**Table 2:** Mean DNA copies of *Rickettsia prowazekii* in peripheral blood and various tissues sampled from doxycycline-treated or non-treated mice with dexamethasone-reactivated typhus infections.

	Mean DNA copies/µL blood or mg tissue (no. mice positive/no. tested)	
Sample	Primary infection treated with doxycycline	Without doxycycline
Blood	24 (1/6)	73 (7/8)
Liver	ND	80 (2/5)
Spleen	ND	910 (2/5)
Lungs	338 (1/6)	276 (2/5)
Brain	ND	374 (2/5)

ND: no DNA detected.

*R. prowazekii* rickettsiae were isolated in L929 cells from blood and tissues of dexamethasone-treated mice. Rickettsiae were identified in Gimenez and immunofluoresence-stained cell culture inoculated with blood or triturates of spleen, liver, lungs, or brain harvested from 4 mice at 3 days following the completion of therapy. Rickettsiae could be seen in L929 cells within 7 to 14 days following inoculation with blood or tissue. No *R. prowazekii* rickettsiae were isolated in cell cultures inoculated with blood or tissue samples harvested from mice 8 days following completion of dexamethasone treatment.

The titers of anti-*R. prowazekii* IgG antibodies in the dexamethasone-treated mice (geometric mean titer = 5570, range = 3200–12800) were significantly greater than the titers observed in mice treated with cyclosporine (geometric mean titer = 696, range = 400–1600) or sham-immunosuppressed mice (geometric mean titer = 696, range = 400–1600). No post-treatment IgM antibodies were detected in dexamethasone-treated mice.

### Effect of Doxycycline on R. prowazekii Reactivation

Antirickettsial therapy (i.e., doxycycline) administered to mice at the time of the primary infection diminished the occurrence of detectable rickettsiae in blood and tissues following treatment with dexamethasone. A transient rickettsemia was identified in only 1 of 6 (17%) doxycycline-treated mice following dexamethasone treatment, significantly less than the frequency of rickettsemia in dexamethasone-treated mice receiving no doxycycline (88%). The blood of the mouse treated with doxycycline contained only 24 DNA copies/µL blood significantly lower than the number of copies observed in dexamethasone-treated mice receiving no doxycycline. No rickettsial DNA was detected in the livers, brains or spleens of dexamethasone-treated mice that received doxycycline during the primary infection.

### β-defensin Expression and Histopathology of Lungs of Mice with Relapsing Rickettsemia

**Figure 1:**
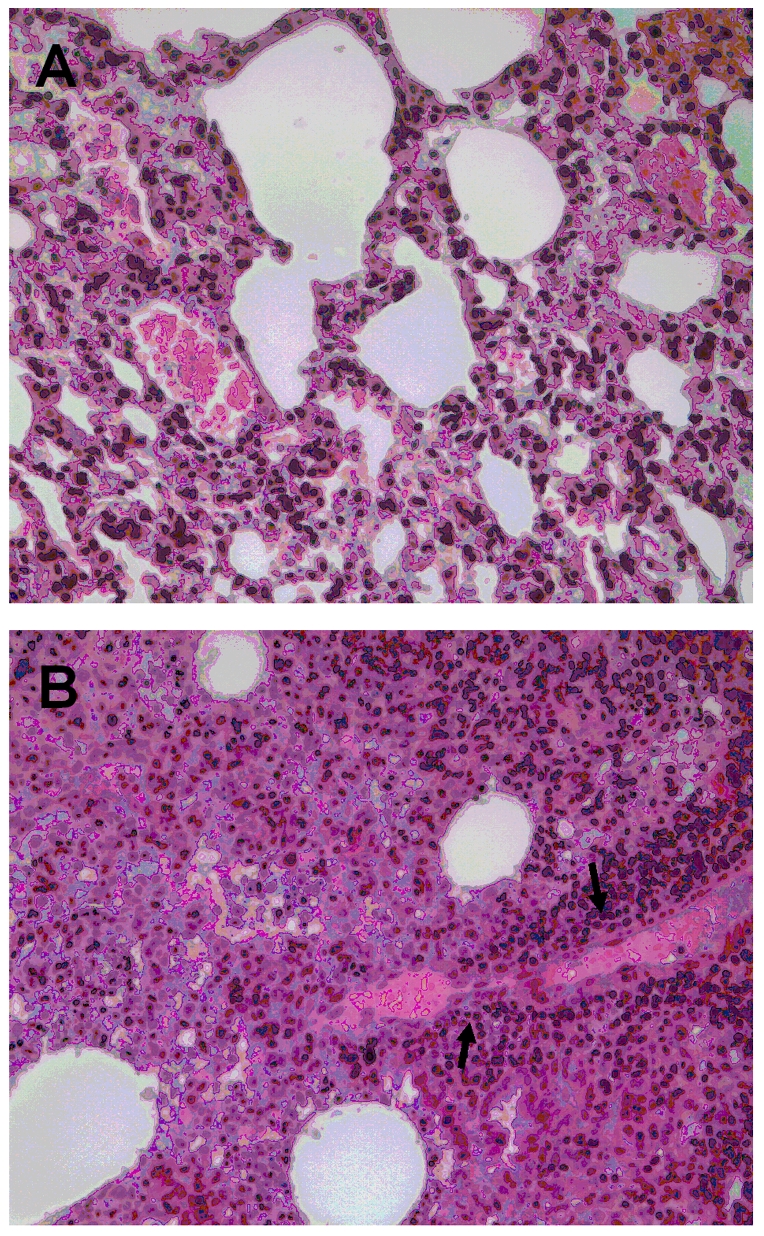
Histopathology of lung lesions in BALB/c mice. Pulmonary parenchyma from mice with reactivated infection, sacrificed 3 days after the end of dexamethasone treatment, showing interstitial inflammatory cell infiltrates comprising predominantly mononuclear leukocytes (A). Pulmonary parenchyma from mice at the time of primary infection (B) showing abundant inflammatory infiltrates and focal vasculitis (arrows). Hematoxylin and eosin stain, original magnifications ×200.

The lungs of dexamethasone-treated mice showed impaired expression of β-defensin transcripts (ratio of expression levels of −8.25±3.7). Multifocal intraalveolar hemorrhages, and interstitial lymphohistiocytic infiltrates were identified in the lungs of mice sacrificed following dexamethasone-reactivated infection (Figure 1A). In general, these lesions were similar to, but less extensive (Figure 1B) than the pulmonary lesions that developed in mice following primary infection with *R. prowazekii* [24]; however, no distinct rickettsial antigens were detected in these lungs by immunohistochemical methods. No histopathologic lesions were identified in the spleens, brains, or livers of any mouse with dexamethasone-reactivated infection.

### R. prowazekii Infection in Cultured Murine Adipocytes and Demonstration of Rickettsial Persistence in Mouse Adipose Tissues

**Figure 2:**
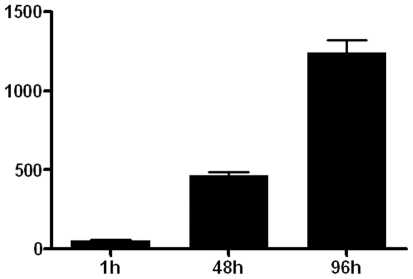
Intracellular replication of *R. prowazekii* in 3T3-L1 adipocytes. Adipocytes were infected with *R. prowazekii* (bacterium-to-cell ratio of 50∶1) for 1 hour, washed and cultured for 2 and 4 days. Infection was measured by immunofluorescence. Results represent mean values (±SD) of 3 experiments.

**Figure 3:**
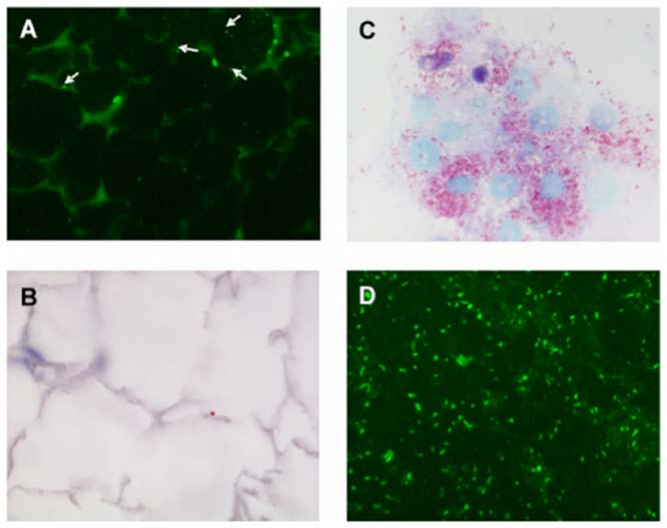
Immunodetection and isolation of *R. prowazekii* from adipose tissues 4 months after primary infection. Immunofluorescence staining showing *R. prowazekii* rickettsiae in adipocytes (arrows), using an anti-OmpB monoclonal antibody (*A*), and rare staining (red-brown) using an immunoperoxidase technique with a polyclonal anti-*R. prowazekii* antibody (*B*). Gimenez (*C*), and immunofluorescence (*D*)-stained preparations of intracellular *R. prowazekii* rickettsiae, isolated from murine adipose tissues in L929 cells. Original magnifications ×400.

By using fluorescence microscopy, *R. prowazekii* rickettsiae were identified in the cytoplasms of differentiated adipocytes cell line 3T3-L1 within 1 hour after exposure to cell-free rickettsiae. Rickettsiae were able to survive and multiply within the murine adipocyte cell line. At 2 days post-infection, approximately 60%–70% of 3T3-L1 cells contained 5–10 rickettsiae, and after 4 days, >90% of the cells contained 10–20 rickettsiae (Figure 2). The persistence of *R. prowazekii* in adipose tissues of mice was assessed by using qPCR, immunostains, and cell culture. Rickettsial DNA was detected in adipose tissues from both of 2 mice sampled at 20 days and at 97 days pi, and from 6 of 11 mice sampled at 120 days pi, while no bacterial DNA was detected in any of the other tissues tested. *R. prowazekii* was also detected by immunofluorescence and immunohistochemistry in sections of fixed adipose tissue from 3 of 6 mice sampled at 120 days pi (Figures 3A, B). Viable *R. prowazekii* rickettsiae were recovered from peritoneal adipose tissues of 3 of 6 PCR-positive mice sacrificed 4 months after the primary infection. Indeed, when L929 cells were inoculated with homogenized peritoneum samples, rickettsiae were detected by Gimenez and immunofluorescent stains in these cultures weeks later (Figures 3C, D). Taken together, our data suggest that adipose tissues may constitute a reservoir for dormant *R. prowazekii*.

## Discussion

Using a recently described murine model for infection with *R. prowazekii* [24], we induced a transient rickettsemia >3 months after the primary infection by long-term treatment with dexamethasone. This reactivation was accompanied by a robust and specific IgG antibody response, without detectable IgM antibodies, similar to the humoral response associated with Brill-Zinsser disease [16], [17], [19], [20], and pulmonary lesions similar to, but generally less extensive than the lesions observed in the lungs of mice following primary infection with *R. prowazekii* [24]. In addition, we were able to detect molecularly and isolate in culture *R. prowazekii* from adipose tissues of mice 4 months following recovery from the primary infection, when no rickettsiae could be detected in lungs, livers, spleens, or brains of these same mice. Collectively these results indicate that adipose tissue serves as a reservoir for recrudescent disease caused by dormant infection with *R. prowazekii*, and suggest that adipocytes are the specific cell type in which dormant rickettsiae reside.

Interestingly, therapy with doxycycline during the primary infection significantly diminished the frequency of rickettsemia in mice following treatment with dexamethasone. If an average bloodmeal consumed by a louse is 0.63 µL [25], it can be estimated, by using data from our study, that approximately 0–15 copies of *R. prowazekii* DNA would be ingested by a louse in each bloodmeal obtained from a doxycycline- treated mouse following reactivation. In contrast, a louse would ingest approximately 46 DNA copies from the blood of mouse that had recovered from the primary infection without treatment with doxycycline. These results suggest that appropriate antibiotic treatment of patients during outbreaks of epidemic typhus will reduce, but may not eliminate, the occurrence of recrudescent disease.

The effect of dexamethasone treatment was specific. Indeed, in our experimental conditions, cyclosporine induced a level of immune suppression (as measured by IFN-γ levels) similar to that induced by dexamethasone; however, cyclosporine was unable to reactivate *R. prowazekii* infection. As cyclosporine acts specifically on T-cell immune responses, it is unlikely that compromise of adaptive immunity by itself is sufficient to initiate Brill-Zinsser disease. In this context, it is possible that dexamethasone initiates recrudescent infection by anti-inflammatory effects on the host's innate immune response [26].

However, it is also possible that long-term treatment with dexamethasone induces biochemical perturbations in adipose tissue that are similar to those triggered by one or more of the exogenous cues that initiate Brill-Zinsser disease, including starvation and extreme social stress. Secretion of endogenous glucocorticoids is increased significantly in starved animals [27], and it is well-recognized that prolonged administration of dexamethasone to animals causes a diminution of fat depots that resemble closely the effects of chronic starvation [28]. More recently, investigators have shown that dexamethasone down-regulates the expression of lipoprotein lipase in vitro and in vivo [29]. Adipocytes obtain most of the lipid needed for triglyceride synthesis by the activity of lipoprotein lipase; dexamethasone alters directly the physiology of these cells and decreases intracellular triglycerides in a manner similar to prolonged caloric restriction. Because dexamethasone also acts on lipid metabolism via the lipoxygenase pathway [30], it is possible that glucocorticoids affect adipocyte physiology through several biochemical pathways.

The physiologic complexity of adipose tissue extends far beyond its role as a depot for triglycerides. Indeed, adipocytes are metabolically active endocrine cells that play a crucial role in energy homeostasis, and provide important immunologic functions [31]. The biochemistry of these cells may be modulated by other hormones, cytokines, and macromolecules, and it is possible that the pathogenesis of rickettsial reactivation from adipose tissue depends on multiple and varied physiological stimuli. By example, tumor necrosis factor-α potentiates the reduction of intracellular lipids in starved adipocytes [32]; interestingly, mice exposed to conditions of social disruption and stress secrete markedly elevated levels of TNF-α [33].

Adipose tissue includes a minority of cell types other than adipocytes, including fibroblasts, macrophages, pericytes, vascular endothelium, and smooth muscle cells [31]. We cannot say definitively that adipocytes are the specific cell that sequesters dormant *R. prowazekii* rickettsiae; however, some or all of these non-adipocyte cells are plentiful in each of the other non-adipose tissues from which no rickettsiae were detected molecularly or isolated in culture months after the primary infection. In this context, we believe that the adipocyte constitutes the principal cellular reservoir for dormant typhus infection in our mouse model. The precise cellular location of dormant *R. prowazekii* rickettsiae in infected human hosts remains unknown. To our knowledge, culture isolation of dormant *R. prowazekii* rickettsiae from humans has been described by only once [13], from inguinal lymph node tissues from 2 of 16 Russian immigrants undergoing abdominal surgery in the United States, presumably many years after the primary infection. Because some adipose tissue often accompanies lymph node specimens excised during surgical procedures, it is possible that adipose tissue, rather than the lymph nodes, provided the source for recovery of rickettsiae. Isolation from humans of other types of rickettsiae, including *Rickettsia rickettsii* (the agent of Rocky Mountain spotted fever) and *Orientia tsutsugamushi* (the agent of scrub typhus), as long as 1 year after clinical recovery, has been reported rarely [21], [34]. *Coxiella burnetii*, the causative agent of Q fever, also persists for long time in mice and the persisting bacteria can be transmitted at least to 2 generations [35].

The evaluation of additional tissues from infected mice, including lymph nodes , bone marrow, or other viscera, may yield other locations with dormant *R. prowazekii* rickettsiae; however, it is likely that adipocytes play a crucial, and perhaps unique role in Brill-Zinsser disease. Adipocytes are know recognized as important reservoirs of other intracellular pathogens that remain dormant for many years after the primary infection, including *Trypanosoma cruzi* [36], *Mycobacterium tuberculosis* [37], and *Coxiella burnetii* (Y. Bechah, unpub. data). A careful inspection of the complex biochemical interactions between adipocytes and various metabolic and immunologic pathways are needed to better characterize the pathogenesis of Brill-Zinsser disease; indeed, the propagation of *M. tuberculosis* in adipocytes depends on lipid metabolism [38].

Pulmonary inflammation was identified only in the lungs of mice with dexamethasone-reactivated *R. prowazekii* infection. In a previous study [24] we demonstrated abundant rickettsial antigens in the lungs of mice following primary infection with *R. prowazekii*. In this investigation, we failed to detect antigens by immunohistochemical methods in the lungs of mice with dexamethsone-reactivated infections; however, pulmonary rickettsial loads following primary disease determined in our earlier study were approximately 5 times greater than the levels detected in the lungs of mice with recrudescent disease. Dexamethasone is known to reduce the basal expression and the production of tracheal antimicrobial peptides (defensins) [39] and enhance the susceptibility to respiratory diseases [40]. This may influence the appearance of inflammatory reactions in lungs by decreasing their antimicrobial activity or interfering with their ability to recruit and activate inflammatory cells.

In conclusion, we describe for the first time a recrudescent form of epidemic typhus in a murine model. In this model adipose tissue represents a reservoir in which bacteria may avoid killing by drugs and host defense mechanisms. This model can be applied to test new therapeutic strategies that eradicate dormant bacteria and prevent the occurrence Brill-Zinsser disease, and provide new insights in the pathogenesis of this disease and the mechanisms that allow *R. prowazekii* to persist in vertebrate hosts.

## Materials and Methods

### Ethics Statement

The mice used in our study were handled according to the rules of Décret N° 87–848 du 19/10/1987, Paris. The experimental protocol have been reviewed and approved by the Institutional Animal Care and Use Committee of the Université de la Méditerranée.

### Bacterial Preparation

*R. prowazekii* strain Breinl (ATCC VR-142) was propagated as recently described [24]. Monolayers of murine L929 cells were infected for 7 days and bacteria were harvested and quantified as previously described [41]. The identity of *R. prowazekii* was confirmed by sequencing of PCR products using *gltA* primers, as previously described [42]. Heat-inactivated *R. prowazekii* rickettsiae were obtained by a 60°C treatment for 30 min.

### Experimental Design

**Figure 4:**
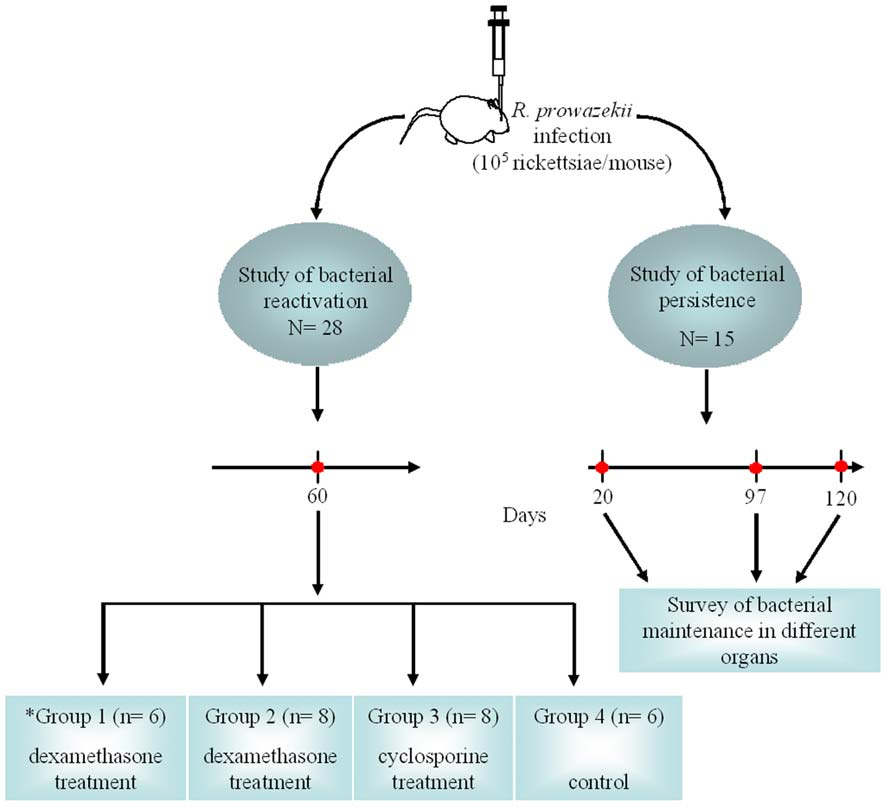
Experimental protocol for study of *Rickettsia prowazekii* persistence and reactivation in BALB/c mice. BALB/c mice were infected intravenously (via the retro-orbital venous plexus) with 1×10^5^ bacteria. Doxycycline was administered to group 1 (*) per os on days 7 and 9. Two months following the primary infection, mice were immunosuppressed with dexamethasone or cyclosporine via the intra-peritoneal route. A control group received sham immunosuppression with phosphate buffered saline. To study bacterial persistence, infected mice were sacrificed at days 20 (n = 2 mice), and 97 (n = 2) post-challenge and the presence of bacteria in different organs was assessed by PCR, cell culture, and immunostaining. Mice sacrificed at 120 days post-infection (n = 11) were infected in a separate experiment and bacterial persistence was evaluated by the same techniques.

7 week-old female BALB/c mice were obtained from Charles River Laboratories. Mice were housed in a biosafety level 3 laboratory and provided sterile food and water ad libitum. Mice were infected with a single dose of 1×10^5^ bacteria injected intravenously (iv) into the retro-orbital plexus [24]. In the first set of experiments, *R. prowazekii*-infected mice were separated into 4 experimental groups (Figure 4). Whole blood specimens were collected by retro-orbital puncture at 3, 6, 9, 30, 60 pi from mice in each group and stored at −20°C for PCR experiments and serum antibody determination (see below). One week post infection, mice from group 1 (n = 6) received a dose of doxycycline (10 mg/kg body weight) per os on days 7 and 9. At 60 days pi, the mice from group 1, and 8 infected but non-treated mice, comprising group 2, were given dexamethasone (160 µg in 200 µL PBS) daily for 30 days by intraperitoneal (ip) injection. Another 8 infected but non-treated mice, comprising group 3, were given cyclosporine (40 mg/kg of body weight) daily for 20 days by ip injection. Six infected but non-treated mice, comprising group 4, were given 200 µL phosphate buffered saline daily for 20 days by ip injection as sham immunosuppression. Mice were sacrificed at days 3 (n = 5) and 8 (n = 3) after the completion of each immuosuppresive treatment. Representative samples of liver, lungs, spleen, and brain were excised aseptically and frozen at −80°C for subsequent PCR assays and cell culture isolation. Samples from each organ were also fixed in 10% formalin for histological and immunohistochemical evaluation.

In a second set of experiments, infected mice were assessed for persistence of *R. prowazekii* at days 20, 97, and 120 pi. Representative samples of liver, lungs, spleen, brain, white adipose tissue from the peritoneum, subcutaneous adipose tissue from the inguinal region, and brown adipose tissue from the dorsal region were excised aseptically at necropsy and frozen at −80°C or fixed in 10% formalin for subsequent PCR assays, inoculation into cell culture and immunostaining.

### IFN-γ Release by Splenocytes

Splenocytes were isolated from treated or non- treated mice to determine the efficiency of immunosuppression. Briefly, splenocytes (2×10^6^ cells/well) were incubated in RPMI1640, containing 25mM HEPES, 10% FBS, 2 mM L-glutamine, 100 U/ml penicillin, and 100 µg/ml streptomycin, in the presence or absence of heat-inactivated *R. prowazekii* (10 rickettsiae per cell) for 24 h [24]. Supernatants were stored at −80°C, and interferon (IFN)-γ levels were determined by immunoassay (R&D Systems; detection limit, 2 pg/mL).

### Blood and Tissue Infection

Bacterial infection was studied by qPCR and shell vial isolation. DNA was extracted from whole blood (200 µL) and tissue samples (25 mg) using the QIAamp Tissue kit (Qiagen). Negative controls consisted of DNA extracts from sham-immunosuppressed mice. DNA was extracted in a final volume of 100 µL. qPCR was performed on 5 µl of blood or tissue DNA extracts using the LightCycler system (Roche Diagnostics) targeting a segment of the rickettsial *gltA* gene [24]. For isolation, tissue samples were first triturated in 2 mL of medium using a sterile scalpel blade. Blood (200 µL) and tissue triturates (500 µL) were inoculated into shell vials containing confluent monolayer of L929 fibroblasts. Rickettsial infection of L929 cells was monitored by using Gimenez staining [43].

### Histopathologic Evaluation and Immunostaining for R. prowazekii

Formalin-fixed, paraffin-embedded tissues were cut at 4-µm and stained with hematoxylin and eosin for histopathologic examination. Sections of peritoneal adipose tissue obtained from mice 4 months pi were also evaluated by immunohistochemical and immunofluorecsence stains using a polyclonal rabbit anti-*R. prowazekii* hyperimmune serum diluted to 1/2000 for immunohistochemistry, or a monoclonal anti-*R. prowazekii* OmpB antibody diluted to 1/500 for immunofluorescence [24].

### Antibody Determination

To determine the titer of circulating antibodies directed against *R. prowazekii*, slides prepared with air-dried, methanol-fixed rickettsiae were incubated with serial dilutions of mouse serum for 30 min. After washing, the bacteria were revealed using labeled goat antibodies directed against IgM or IgG (Beckman Coulter) at a 1∶100 dilution for 30 min. The slides were then washed and examined using a Zeiss fluorescence microscope [24].

### Reverse Transcription-PCR (RT-PCR) for β-defensin

RT-PCR for β-defensin was carried out as previously described [24]. Briefly, total RNA was isolated from lung tissue using a Qiagen kit. cDNA synthesis was obtained by using an oligo(dT) primer and M-MLV reverse transcriptase (Invitrogen). PCR was performed using the cDNA targeting β-defensin 1(Defb1) with the following primers (forward and reverse): *Defb1*, 5′-CCAGCTGCCCATCTAATACC-3′ and 5′-AATCCATCGCTCGTCCTTTA-3′. β-actin used as control, F5′-TGGAATCCTGTGGCATCCATGAAA-C-3′, R5′-TAAAACGC-AGCTCAGTAACAGTCCG-3′. PCR parameters were: denaturation at 94°C for 3 min, followed by 45 cycles of 94°C for 30 s, annealing at 54°C and final extension at 72°C for 1 min. The fold change (FC) in expression of *Defb1* relative to β-actin was determined as follows: FC = 2^−ΔΔCt^ where ΔΔCt = (Ct_Target_−Ct_Actin_)_sample_−(Ct_Target_−Ct_Actin_)_reference_, with the sample values represented by tissues from dexamethasone-treated mice and the reference values as tissues from non-treated mice.

### In Vitro Propagation of R. prowazekii in Cultured Murine Adipocytes

Adipocytes were prepared as previously described [37]. Briefly, murine 3T3-L1 fibroblasts (ATCC, Manassas, VA) were seeded on 12-mm diameter glass coverslips in 24 well-plates at 4×10^4^ cells/well, and incubated in Dulbecco's modified Eagle's medium (DMEM) (Invitrogen) containing 10% FCS and 2 mM L-glutamine. When cells were confluent, adipocyte differentiation was induced by adding 100 mM 3-isobutyl-1-methylxanthine (IBMX), 250 nM dexamethasone and 1 µg bovine insulin. After 2 days, the medium was replaced with DMEM containing 10% FCS and 1 µg insulin. Differentiated adipocytes were infected with rickettsiae after an additional 3 days. Uptake of *R. prowazekii* by adipocytes and the intracellular fate of rickettsiae were studied as follows. Cells were incubated with *R. prowazekii* (50 rickettsiae per cell) for 1 h at 37°C and unbound bacteria were removed by extensive washing. Cells were then incubated with DMEM containing 10% FCS and L-glutamine. Bacterial replication in differentiated 3T3-L1 cells over time was determined over time by immunofluorescence staining. Cell cultures were fixed on glass slides in 3% paraformaldehyde, permeabilized with 0.1% Triton X-100, and stained by using a monoclonal anti-*R. prowazekii* OmpB antibody diluted at 1/500. Stained slides were mounted with Mowiol and the cells were examined using epifluorescence microscopy. The percentage of infection was calculated as 100× the product of the mean number of rickettsiae per infected cell and the percentage of infected cells.

### Statistical Analysis

Statistical analyses were performed using GraphPad Prism, software 4.0. Quantitative data were compared with the Mann-Whitney *U* test or Fisher test. *P* values<0.05 were considered significant.

The findings and conclusions described herein are those of the authors and do not necessarily represent the views of the U.S. Department of Health and Human Services.
