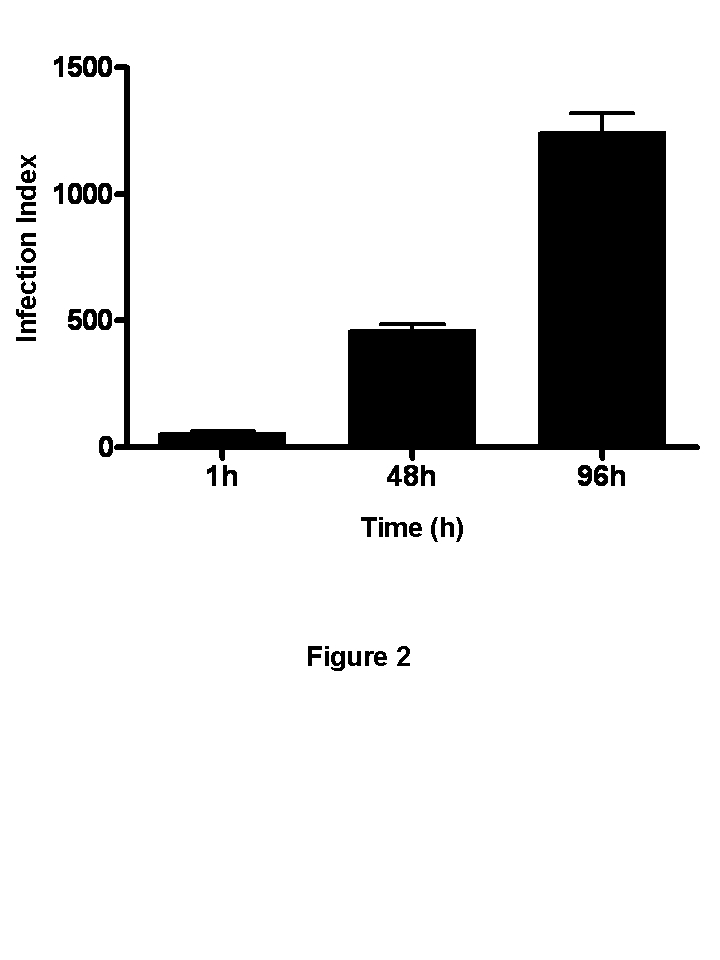# Correction: Adipose Tissue Serves as a Reservoir for Recrudescent *Rickettsia prowazekii* Infection in a Mouse Model

**DOI:** 10.1371/annotation/79b2d8c8-ec9e-484c-8420-cdb64fb72f1c

**Published:** 2010-01-21

**Authors:** Yassina Bechah, Christopher D. Paddock, Christian Capo, Jean-Louis Mege, Didier Raoult

The x-axis and y-axis labels were omitted from Figure 2. Please view the corrected figure at: 

**Figure:**